# EBI metagenomics—a new resource for the analysis and archiving of metagenomic data

**DOI:** 10.1093/nar/gkt961

**Published:** 2013-10-26

**Authors:** Sarah Hunter, Matthew Corbett, Hubert Denise, Matthew Fraser, Alejandra Gonzalez-Beltran, Christopher Hunter, Philip Jones, Rasko Leinonen, Craig McAnulla, Eamonn Maguire, John Maslen, Alex Mitchell, Gift Nuka, Arnaud Oisel, Sebastien Pesseat, Rajesh Radhakrishnan, Philippe Rocca-Serra, Maxim Scheremetjew, Peter Sterk, Daniel Vaughan, Guy Cochrane, Dawn Field, Susanna-Assunta Sansone

**Affiliations:** ^1^European Molecular Biology Laboratory, European Bioinformatics Institute (EMBL-EBI), Wellcome Trust Genome Campus, Hinxton, CB10 1SD, UK, ^2^Oxford e-Research Centre, University of Oxford, 7 Keble Road, Oxford, OX1 3QG, UK and ^3^NERC Centre for Ecology and Hydrology, Maclean Building, Benson Lane, Crowmarsh Gifford, Wallingford, OX10 8BB, UK

## Abstract

Metagenomics is a relatively recently established but rapidly expanding field that uses high-throughput next-generation sequencing technologies to characterize the microbial communities inhabiting different ecosystems (including oceans, lakes, soil, tundra, plants and body sites). Metagenomics brings with it a number of challenges, including the management, analysis, storage and sharing of data. In response to these challenges, we have developed a new metagenomics resource (http://www.ebi.ac.uk/metagenomics/) that allows users to easily submit raw nucleotide reads for functional and taxonomic analysis by a state-of-the-art pipeline, and have them automatically stored (together with descriptive, standards-compliant metadata) in the European Nucleotide Archive.

## INTRODUCTION

Ever since the term ‘metagenome’ (meaning ‘the collective genomes of […] microflora’) was coined by Handelsman *et al.* in 1998 (1), biologists have been eager to tap into the potential of previously uncultured organisms and understand their impact on a variety of environments. At that time, however, sequencing metagenomes was expensive, due to a reliance on Sanger sequencing. This low-throughput sequencing approach relies on clone libraries generated from cultured organisms; a laborious process that proves to be problematic when samples cannot easily be cultured in the laboratory (as is often the case with environmental samples). With the advent of next-generation sequencing (NGS) technologies, sequencing costs have dropped sharply and metagenome sequencing has become a reality. As a result, scientists are able to directly and comprehensively sample the genetic makeup of entire communities of microbes for the first time, without the need for culturing. Metagenomic studies have been carried out on a multitude of environments, including body sites [e.g. cow rumen (2), human intestinal tract (3) and oral cavity (4)], marine (5), freshwater (6), soil (7) and air (8). Recently, the expense has shifted away from sequence data generation toward the analysis and management of the extremely large data sets produced by NGS machines.

To ameliorate this problem for end users, European Molecular Biology Laboratory-European Bioinformatics Institute (EMBL-EBI) committed to creating a resource that built on existing EMBL-EBI infrastructure and was specifically targeted at metagenomic researchers: EBI metagenomics (http://www.ebi.ac.uk/metagenomics/). Several other resources exist that already offer analysis of metagenomic data, such as Metagenomics-Rapid Annotations using Subsystems Technology (MG-RAST) (9), Community Cyberinfrastructure for Advanced Microbial Ecology Research and Analysis (CAMERA) (10) and Integrated Microbial Genomes and Metagenomes (IMG/M) (11). However, until the advent of EBI metagenomics, there was no such resource in Europe. Data are initially submitted to and archived in the European Nucleotide Archive (ENA), which has the mandate to hold public data for long-term stewardship and enable future reuse by the community. It is then processed by EBI metagenomics, where it undergoes quality-control checks, and functional and taxonomic analyses. The system has been designed with the potential to deal with metatranscriptomics, metaproteomics and metametabolomics data in the near future. Being housed at the EBI, there is ample potential for data integration with a range of other in-house bioinformatics resources.

## DATA SUBMISSION

Users can submit data to EBI metagenomics using a variety of tools with graphical user interfaces or programmatic interfaces. Data submitted to the system can be held privately for a time (for example, during the prepublication period) until the researcher wishes to make it public. Submitters requesting prepublication confidential hold for their data are allowed up to 2 years under this status.

Users are required to register for an account, which is used to transfer raw sequence and associated metadata for archiving and analysis. If the study and/or sample have not yet been made public, the user will also need to log in to see the analysis results for that data set. Once a study/sample is public, the raw data and associated results are free to browse by the public through the EBI metagenomics web interface.

### Accepted/required data types

A major aim in the development of this resource has been to encourage metagenomics researchers to openly share their data as widely as possible, and to also describe their data in sufficient detail such that other scientists are able to extract maximum value from it. For this reason, metadata (for example, describing the sample that a metagenome was sequenced from and the protocols used) are expected to meet minimum standards. These are defined by the Genomic Standards Consortium (GSC) (12) through the provision of standards to specifically describe metagenomic and marker gene-based data sets (MIGS/MIMS and MIMARKS), as part of the wider MIxS (minimum information about any sequence) standard (13).

The structure of data in EBI metagenomics partially mirrors the way that the ENA organizes data objects, with one project containing one or more samples. Each sample can have one or more experiments associated with it (e.g. genomic, transcriptomic), and each experiment may contain one or more runs from a sequencing machine. The use of projects to group samples together is done at the discretion of the submitter. Raw sequence reads from all major NGS platforms are accepted by the resource, including Roche/454 (Roche Diagnostics Corp.) and Illumina (Illumina Inc.). At present, projects consisting solely of marker gene/amplicon-based data sets (e.g. 16S surveys of an environment) will only be analyzed using the metagenomics pipeline if they are part of a larger study including shotgun-generated metagenomic data.

### Use of ENA Webin

**Figure 1.**
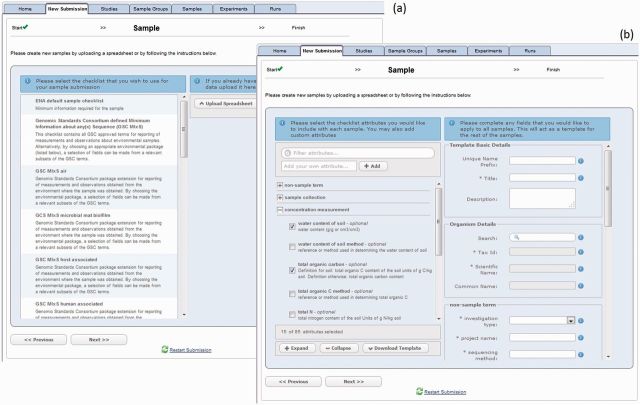
Screenshots from the ENA’s Webin tool showing the pages where (**a**) users select which MIxS checklist they wish to use for the submission and (**b**) the subsequent list of optional and mandatory fields they can enter values for. Note that a spreadsheet may optionally be used to perform the same task.

Submission of metagenomics data may take place using the interactive web submission tool, Webin (14) (http://www.ebi.ac.uk/ena/submit/sra/#home). This tool leads the submitter through a process in which they authenticate (or register if they are not already submitters), provide outline information about their study, describe their samples and, finally, upload sequence read data. Steps for sample description (see Figure 1) have been developed in the Webin framework in support of MIxS compliance and offer flexible spreadsheet upload and error reporting to submitters.

### Use of Investigation/Study/Assay tools

Complementary to ENA Webin, the Investigation/Study/Assay (ISA) open-source software suite and metadata tracking framework (15) may also be used to submit metagenomic data. ISAcreator, part of the ISA tool suite (http://isa-tools.org), assists researchers in collecting and curating metagenomics data sets at source (following the GSC minimum information requirements); visualizing, managing and storing it locally before repository submission; and reformatting it, producing XML files compatible for submission to the ENA. ISA also facilitates publication of data sets in an increasing number of data publication journals, such as BioMedCentral (http://www.biomedcentral.com), GigaScience (http://www.gigasciencejournal.com/) and Nature Publishing Group’s Scientific Data (http://www.nature.com/scientificdata/); the latter two are directly using the ISA framework. GSC-compliant configurations for the ISA tools are readily available for download, along with the latest version of the software at http://github.com/ISA-tools. The ISA framework is designed for and used in an increasingly diverse set of life science domains, including metabolomics, proteomics, system biology, environmental health, environmental genomics and stem cell discovery (16). It currently powers the EBI’s MetaboLights repositories (17) and is well placed to allow submission of multi-assay studies (e.g. metatranscriptomics, metaproteomics and metametabolomics) to EBI metagenomics in the future.

### Web Service-based submission

Metagenomics data submissions can be fully automated, which is an option typically used by larger-scale sequence facilities. Data files to be submitted are transferred into a secure upload area and a number of distinct metadata objects are prepared. Finally, a REST call draws on metadata objects and data files according to a requested transaction (e.g. use of the ‘VALIDATE’ action would validate the data in the files and the ‘ADD’ action would upload and submit them to the resource).

### Current data content

At the time of writing (26 September 2013), the EBI metagenomics resource contains information about 1189 samples in 45 studies.

## ANALYSIS PIPELINE

**Figure 2.**
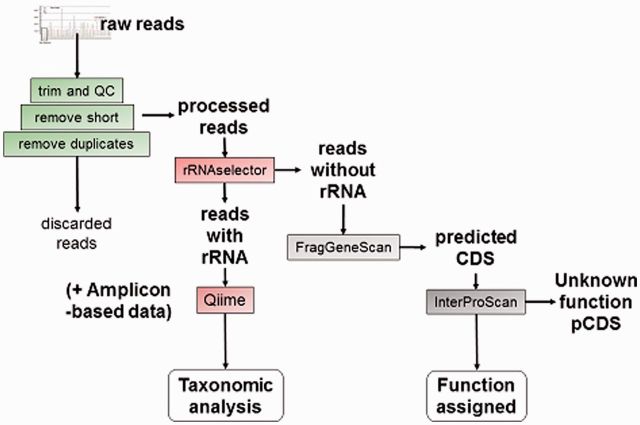
Overview of the pipeline used by EBI metagenomics to process raw sequence files and predict the functions and taxa present in a given sample.

Once raw reads are received, they are queued for analysis through EMBL-EBI’s metagenomics pipeline. Studies and samples that are annotated to minimum standards are prioritized. The analysis pipeline currently consists of quality control (QC), feature (RNA and protein) prediction, function prediction and taxonomic prediction steps (see Figure 2). The pipeline is fully automated and has a Python framework that manages the execution of individual steps on a compute cluster via IBM Platform Load Sharing Facility (LSF; http://www-03.ibm.com/systems/technicalcomputing/platformcomputing/products/lsf/) and collates results files together in a simple directory structure. In its current format, the pipeline is optimized to the EBI LSF configuration and therefore not readily adaptable to other platforms. However, the pipeline is currently being ported to run on the Taverna Workflow Management System (18), to make it more modular, flexible and render the source code easy to share with the wider metagenomics community. Results are summarized and made available for download via the EBI metagenomics web interface (see ‘Data Access’ section).

### Quality control

Different QC steps are performed, depending on the sequencing platform used. Fundamentally, QC steps are intended to remove low-quality and uninformative reads from the data set, so that they are not needlessly passed on to the later stages of the pipeline. The QC steps include read trimming [to remove adapters, etc. using BioPython sff-trim (19) and trimmomatic (20)], removal of ambiguous leading/trailing bases, removal of reads shorter than 100 nucleotides, removal of reads where the proportion of ambiguous bases ≥10%, clustering to remove duplicate sequences [using UCLUST v1.1.579 (21) or using pick_otus.py from QIIME v1.5 (22) for prefix-based filtering] and repeat masking using RepeatMasker open-3.2.2 (http://www.repeatmasker.org). Note that Illumina paired-end reads are merged together using SeqPrep (http://github.com/jstjohn/SeqPrep) before being submitted to the pipeline.

### Feature prediction

The pipeline predicts both ribosomal RNA coding [using models from rRNAselector v1.0.0 (23)] and protein coding [using FragGeneScan v1.15 (24)] features. Predicted rRNAs are used for taxonomic analysis, particularly in the absence of marker gene studies for the same sample, and predicted protein coding sequences (pCDS) are fed into the functional analysis steps in the pipeline.

### Function prediction

Function prediction is performed by analyzing the predicted coding sequences using InterProScan 5 (25). Not all of InterPro’s member databases are designed to work on fragmentary data or microbial sequences, and therefore, only a subset of the 11 available InterPro database analyses are run. These are CATH-Gene3D v3.5.0 (26), PRINTS v41.1 (27), Pfam v24.0 (28), TIGRFAMs v9.0 (29) and PROSITE Patterns v20.66 (30). Gene Ontology (GO) terms (31) for molecular function, biological process and cellular component are associated to the pCDSs by virtue of the InterPro2GO mapping service (32).

### Taxonomic prediction

QIIME v1.5 is used to classify reads into operational taxonomic units (OTUs), to give an indication of the diversity of species found in a particular sample. Presently, we use the Ribosomal Database Project classifier (33) and the Greengenes reference database (34) for classification of archaeal and bacterial species, but it is our intention to expand this to include nonbacterial marker genes, such as 18S rRNA and internal transcribed spacer sequences in the near future.

## DATA ACCESS

The complete set of information about a metagenome, including the raw, submitted sequence data, descriptive metadata and analysis results is made freely available via the EBI metagenomics web interface, once the owner of the data has made it public. The most recently submitted public projects and samples are listed on the home page; if a user is logged in, they will see their privately held projects and samples instead. The home page also lists up-to-date statistics about the content of the database and recent news/events on the right hand side of the page.

There is a horizontal menu below the banner on each page, which allows a user to switch between different sections of the site (for submitting data, browsing projects, browsing samples, learning about EBI metagenomics and contacting the team). On the pages for browsing samples and projects, a user may enter a search term into the search box and only those projects or samples containing that term in their name or description will be displayed.

Each project has an overview page that displays the information supplied by the submitter to describe the project, including a link to related publications (where appropriate), and primary contact details. At the bottom of the project page is a list of all samples associated with that project, and links to the analysis results for each.

The sample pages have multiple sections that can be accessed via tabs at the top. If an analysis has not yet completed (i.e. no results files are available), the tab will be grayed out, indicating that the section currently does not contain data.

The ‘overview’ section shows all metadata that has been submitted to describe the sample. A subset of metadata, such as the general description, the latitude and longitude, or the species that the sample is taken from, is highlighted. A full table of all metadata that has been submitted is also shown.

The ‘quality control’ (QC) tab contains a chart of the number of sequences present after each step in the QC process (e.g. the number of reads initially submitted to the pipeline, or the number remaining after sequences shorter than 100 nucleotides are removed).

**Figure 3.**
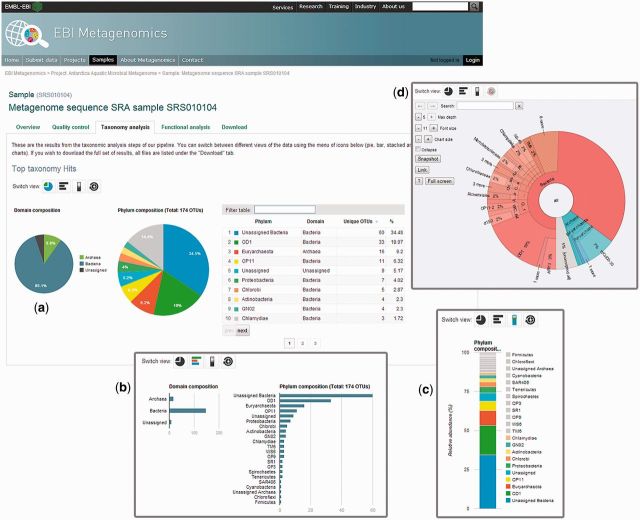
Screenshots of the analysis results pages where users can visualize the outputs of the analysis pipeline in a number of formats. A pie chart (**a**) summarizing the OTUs present in the sample is shown by default on the taxonomy results page and users can switch between a bar chart (**b**), stacked chart (**c**) and interactive Krona viewer (**d**) by selecting an icon from the ‘switch view’ menu.

The ‘taxonomy analysis’ tab gives users a number of different ways of visualizing the results of taxonomic analysis of their sample (see Figure 3). The page uses Google’s chart API (http://developers.google.com/chart/) to display information in simple pie, bar or stacked bar charts, each with a tabular representation of the data to the right side of the page. The Krona viewer (35) is also embedded as a fourth option, to allow interactive browsing of the data. Users can alternate between these different visualization options by clicking on the icons in the menu immediately above the content.

The ‘function analysis’ tab displays a breakdown of the types of sequences predicted, and uses the Google API to display the top InterPro matches in a pie chart, with an accompanying data table. Beneath the InterPro results, the GO terms describing the sample are summarized using a GO slim chart that has been developed specifically for metagenomics samples. The metagenomics GO slim is available via the GO Web site (http://www.geneontology.org/GO_slims/goslim_metagenomics.obo).

Finally, all of the intermediate and final results files generated by the pipeline may be downloaded in a variety of formats from the ‘Download’ tab, for use in compatible tools.

## COMPARISON WITH AND LINKS TO OTHER PUBLIC METAGENOMICS RESOURCES

MG-RAST, CAMERA and Integrated Microbial Genomes and Metagenomes (IGM/M) are currently considered to be state-of-the-art resources for analysis of metagenomics data. EBI metagenomics represents an additional complementary analysis resource that builds on EBI expertise in sequence data archiving and analysis. Uniquely, its close integration with the ENA means that users can be confident that their data are stably archived, and receives accession numbers—a prerequisite for many publications. Provision of the Webin and ISA tools streamlines the submission process and ensures that all required data are captured and described appropriately. The InterPro-centric approach to functional analysis, meanwhile, provides a powerful and sophisticated alternative to BLAST-based approaches, and use of the GO to annotate results means they are directly comparable with data in other resources that use GO terms for annotation, such as UniProtKB (36).

All of the resources (MG-RAST, CAMERA, IGM/M and EBI metagenomics) have representatives in the GSC and have all adopted and implemented the MIxS checklists. Having uniform sample descriptions is a step toward achieving interoperability between the different resources. There is an effort to establish a platform for next-generation collaborative computational infrastructures, called M5 (Metagenomics, Metadata, MetaAnalysis, Models and MetaInfrastructure) in which all parties are involved (37).

## FUTURE PLANS

A number of new features for the resource are currently in development, as described below.

### Data submission improvements

Future submissions-related work includes Webin interface improvements to capture MIxS information more intuitively, to provide deeper within-field support cases where multiple pieces of information are combined (e.g. links to physical locations of preserved samples) and increased granularity in submitted MIxS fields. Enhancements to the underlying ISA code, interface and documentation to facilitate high levels of standards-compliant data submission to ENA and the EBI metagenomics resource in particular are also planned. The ENA validation mechanisms will be improved to give more informative and intuitive error reporting. ISA will be further customized to enable interoperability between the SRA XML converter and the improved ENA programmatic submissions API.

## Metadata searching

The search engine used to filter the lists of projects and samples is currently basic. We intend to create a more powerful engine that will give users more control over the precise fields that they are able to search. We will initially focus on fields that are part of the minimum standards for metagenomes and describe the samples or experiments. Eventually we intend to allow users to search on additional, nonmandatory metadata fields and also query analysis results (e.g. ‘show me all soil samples where function X is present’).

### Results visualization (and comparison)

We plan to add a number of useful visualizations and analysis results to the web interface, including rarefaction curves and alpha/beta diversity calculations. Information about pathways associated with the sample by virtue of the proteins’ InterPro hits will be added, as well as the ability to visualize these data in a pathway viewer. We will also develop a tool for comparing multiple samples to each other (for example, results across a time-series experiment). This will necessitate normalizing the data before comparison and providing results in formats compatible with different visualization tools.

### Web service access

We plan to include web services for EBI metagenomics to allow people to programmatically access the data and analysis results that are contained within the resource.

### Improved multi-omic support

Presently, the site is focussed mainly on shotgun metagenomes; however, we are aware that an increasing number of scientists are performing multi-omic experiments (e.g. proteomics, metabolomics) on community samples. The use of the ISA framework facilitates the submission and representation of multi-omic experiments in EBI metagenomics; it has been designed with this use case in mind and is already being used in such scenarios in other domains. We also plan to alter the web interface in the near future so that if (for example) a marker gene study, genomic and transcriptomic experiments were performed on the same sample, the analysis results from these different experiments would be displayed in the same sample context.

The development of EBI metagenomics is user-driven and we welcome feedback and ideas via the contact form linked from the main navigation menu on the Web site.

## CONCLUSIONS

The EBI metagenomics resource has been publicly available since December 2011. Through it, researchers are able to submit standards-compliant metagenomics data sets and have them analyzed and archived for public reuse. For convenience, users may submit data using a variety of tools, including the ISA tool suite and ENA’s Webin tool, as well as via web services. Recently, a fuller set of analyses and visualization tools have been made available to the public and these will be added to over the coming months to enhance users’ abilities to access and interpret their data more easily.

## FUNDING

The Biotechnology and Biological Sciences Research Council [BB/I02612X/1 to S.H., BB/I025840/1 and BB/I000771/1 to S.A.S.]; European Commission’s Seventh Framework Programme for Research [Joint Call OCEAN.2011-2: Marine microbial diversity—new insights into marine ecosystems functioning and its biotechnological potential; 287589 to S.H.]; University of Oxford e-Research Centre (to S.A.S.); European Molecular Biology Laboratory (to S.H.). Funding for open access charge: European Molecular Biology Laboratory.

*Conflict of interest statement*. None declared.
